# Transcriptomic Profiling Reveals Divergent Immune Responses to AAV1 and AAV-ie in Mice Inner Ear

**DOI:** 10.7150/ijms.121060

**Published:** 2026-01-08

**Authors:** Dazhi Shi, Lei Han, Can Li, Guannan Geng, Luoying Jiang, Xiaoyun Chen, Fengzhao Yang, Yong Feng, Junli Luo, Yilai Shu

**Affiliations:** 1 Department of Otorhinolaryngology, The Second Affiliated Hospital, Hengyang Medical School, University of South China; Hengyang, 421001 China.; 2 ENT Institute and Department of Otorhinolaryngology, Eye & ENT Hospital, Fudan University, Shanghai 200031, China.; 3 Shanghai Key Laboratory of Gene Editing and Cell Therapy for Rare Diseases, Fudan University; Shanghai 200031, China.; 4 State Key Laboratory of Brain Function and Disorders and MOE Frontiers Center for Brain Science, Fudan University; Shanghai, 200032, China.; 5 Institutes of Biomedical Sciences, Fudan University; Shanghai 200032, China.; 6 NHC Key Laboratory of Hearing Medicine, Fudan University; Shanghai, 200031, China.; 7 Department of Otolaryngology, The First Affiliated Hospital of Nanchang University; Nanchang, 330006, China.; 8 Department of Otorhinolaryngology, The Affiliated Changsha Central Hospital, Hengyang Medical School, MOE Key Lab of Rare Pediatric Diseases & Institute for Future Sciences, University of South China; Changsha, 410028, China.

## Abstract

Adeno-associated virus (AAV) unequivocally emerges as one of the most powerful and promising delivery vectors for gene therapy targeting hereditary hearing loss. Following AAV transduction in the inner ear, varying degrees of natural immune responses are triggered, primarily characterized by macrophage activation and the secretion of pro-inflammatory factors. Additionally, the production of neutralizing antibodies may affect the efficacy of gene therapy. To evaluate immune dynamics, we injected AAV1 and AAV-ie (capsids with distinct transfection efficiencies) into murine cochleae and analyzed temporal transcriptomic profiles. Our results demonstrate that both capsids induce immune activity but with critical temporal and intensity differences that AAV1 elicits significantly later and milder immune reactions compared to AAV-ie. These findings establish that dynamic cochlear gene expression profiles directly inform the selection of immunologically optimized AAV vectors to minimize adverse responses in future hereditary hearing loss gene therapies.

## 1. Introduction

Approximately 466 million people worldwide suffer from disabling hearing loss, including 34 million children, according to data from the World Health Organization 1. Traditional treatment options, such as hearing aids and cochlear implants, can only provide partial improvements in hearing and do not address the underlying causes of the issue. However, recent advancements in gene therapy technology, particularly adeno-associated virus (AAV) vectors, have shown promising potential for treating hereditary deafness 2, 3.

Gene therapy facilitated by the AAV has shown considerable promise in reinstating hearing capabilities to address genetic hearing impairment, extending beyond just mouse models 4, but also in clinical trials 5-7. The inner ear is an optimal target for AAV delivery due to its anatomical isolation and the high transduction efficiency of AAVs in key cell types within the cochlea, particularly in hair cells and supporting cells 8. Although most clinical trials and preclinical studies have confirmed the safety of AAV delivery in the inner ear 5, 9, emerging evidence suggests that AAVs can elicit both innate and adaptive immune responses in mammalian systems, potentially compromising therapeutic efficacy 10.

The mammalian cochlea, traditionally considered immune-privileged due to its blood-labyrinth barrier and limited immune surveillance, harbors resident macrophages that regulate local immune homeostasis 11. The mammalian cochlea's immediate immune reactions have been identified in various pathological states, encompassing noise injuries, cochlear implantation, ear toxicity, and infections 12. Under such circumstances, activated macrophages demonstrate the phagocytic removal of impaired spiral ganglion neurons or hair cells 12. In addition, macrophages facilitate synaptic repair following moderate noise exposure, preserving hair cell integrity while mediating selective ribbon synapse loss 13, 14. Furthermore, noise- or infection-induced inflammation in filtrates in the inner ear predominantly comprises monocytes/macrophages, as highlighted in studies of cochlear immune reactivity 15. Recent studies further reveal that AAV transduction activates immune pathways in a serotype- and promoter-dependent manner 16. For instance, AAV9 induces stronger macrophage infiltration and prolonged pro-inflammatory cytokine release compared to AAV1, while CMV-driven constructs provoke more robust immune activation than CBA-regulated counterparts 16.

However, the molecular mechanisms underlying AAV-triggered immune responses in the inner ear remain poorly characterized. While transient cytokine release (e.g., IL-6, TNF-α) and macrophage recruitment have been observed post-AAV administration 16, systemic analyses of immune-related gene expression profiles are lacking. Furthermore, the extent to which adaptive immune responses - such as neutralizing antibody production or T cell activation, impact long-term transgene expression in the cochlea remains unclear 17. It is essential to address these questions to optimize AAV vector design and delivery strategies to balance transduction efficiency with immune tolerance.

In this study, we systematically investigate transcriptome change elicited by AAV1 and AAV-ie 18 following intracochlear delivery in neonatal mice. Through auditory brainstem response (ABR) assessments, and immunohistochemistry, we confirmed the safety of both AAV1 and AAV-ie transduced in the cochlea, and through deep sequencing, we identified serotype-specific differences in immunogenicity elicited by AAV administration and mapped temporal dynamics of immune-related gene expression. Bioinformatics analysis was conducted to assess the functions and pathways of differentially expressed genes (DEGs) related to immune responses from AAVs. Key DEGs between AAV1 and AAV-ie administration were confirmed via qRT-PCR. Our findings provide critical insights into the immunological barriers of inner ear gene therapy and inform the rational selection of AAV components for clinical translation in future.

## 2. Materials and Methods

### 2.1 Animal models

ICR wild-type mice, aged 2 to 3 days, were utilized in this study. The mice were randomly assigned to various experimental groups, ensuring that each group contained at least three subjects. All experimental protocols received approval from the Institutional Animal Care and Use Committee of Fudan ENT (Protocol #202107004S).

### 2.2 AAV vector construction

The creation of AAV vectors in HEK293 cells was achieved through a conventional polyethylenimine (PEI)-based triple-plasmid co-transfection technique. In the transfection process, the CAG-NLS-tdTomato-WPRE-SV40polyA plasmids, pHelper, and their respective pRep2Cap capsid plasmids were utilized, maintaining a molar ratio of 1:1:1. Engineered AAV vectors were gathered from the cell pellets as well as the media. The rAAVs extracted from the cellular pellets underwent a threefold freeze-thaw procedure. Polyethylene glycol (PEG)/NaCl was employed to precipitate the rAAVs present in the media. qRT-PCR was employed to ascertain the concentration of the unprocessed AAV product in each collected AAV serotype. The viral particles were preserved at a temperature of -80°C until they were needed.

### 2.3 Intracochlear injection

In the experiment, postnatal 2-3 mice were subjected to hypothermic anesthesia, with the exposure time in the ice bath carefully controlled to last between 2 and 3 minutes. A precise incision was made behind the ear to access the round window membrane (RWM). One µL of AAV-ie or AAV1 vector solution (1×10^13^ VG) was injected at a rate of 8 nL/s using a Nanoliter 2000 Injector (WPI, USA). Following the completion of the injection, a 6-0 single-strand nylon suture was used to close the skin wound. Standard postoperative care was provided after the procedure.

### 2.4 Auditory Brainstem Response (ABR)

Recordings of ABRs were conducted with a TDT BioSigRP system (Tucker-Davis Technologies, Alachua, FL) in an acoustically insulated chamber, four weeks post intracochlear injection. The mice received intraperitoneal anesthesia using xylazine (10 mg/kg) and ketamine (100 mg/kg) and were then positioned on a heating pad. A trio of needle electrodes was placed beneath the skin: recording in the pinna, reference between the ears, and ground on the rump. The animals underwent examinations for otitis media and accumulation of cerumen, deliberately omitting any ears impacted in the study. Acoustic stimuli in tone bursts were delivered in reductions of 5 dB, ranging from 90 dB to 20 dB SPL, across frequencies of 4, 8, 16, 24, and 32 kHz. The threshold for ABR was identified as the minimal SPL where the ABR wave was discernible beyond the ambient noise, as ascertained by a pair of separate observers.

### 2.5 Immunofluorescence staining

Cochleae harvested two weeks post-injection were fixed in 4% paraformaldehyde, decalcified in 10% EDTA, and micro-dissected into basal, middle, and apical turns. Subsequently, the tissues were subjected to immunostaining with rabbit polyclonal Myosin7a (1:500 dilution, Proteus Biosciences, Ramona, CA, USA) and goat anti-Sox2 (1:500 dilution, Biolegend, San Diego, CA, USA) as primary antibodies. After being washed three times with PBS, secondary antibodies labeled with fluorescence-donkey anti-rabbit IgG Alexa Fluor 488 and donkey anti-goat IgG Alexa Fluor 647 (diluted 1:500, Thermo Fisher Scientific, MA, USA) were incubated in the dark for two hours at room temperature. The nuclei were counterstained with DAPI (#P36962, Thermo Fisher Scientific, ma, usa).

### 2.6 imaging and Analysis

Samples were prepared and images of fluorescent z-stack confocal microscopy were captured with a laser scanning confocal instrument. A complete image of the cochlea was recreated using Adobe Photoshop and ImageJ software. Transduction efficiency was quantified as the percentage of Myosin7a+/tdTomato+ hair cells and Sox2+/tdTomato+ supporting cells across three cochlear turns.

### 2.7 RNA sequencing and functional enrichment

**Figure 1:**
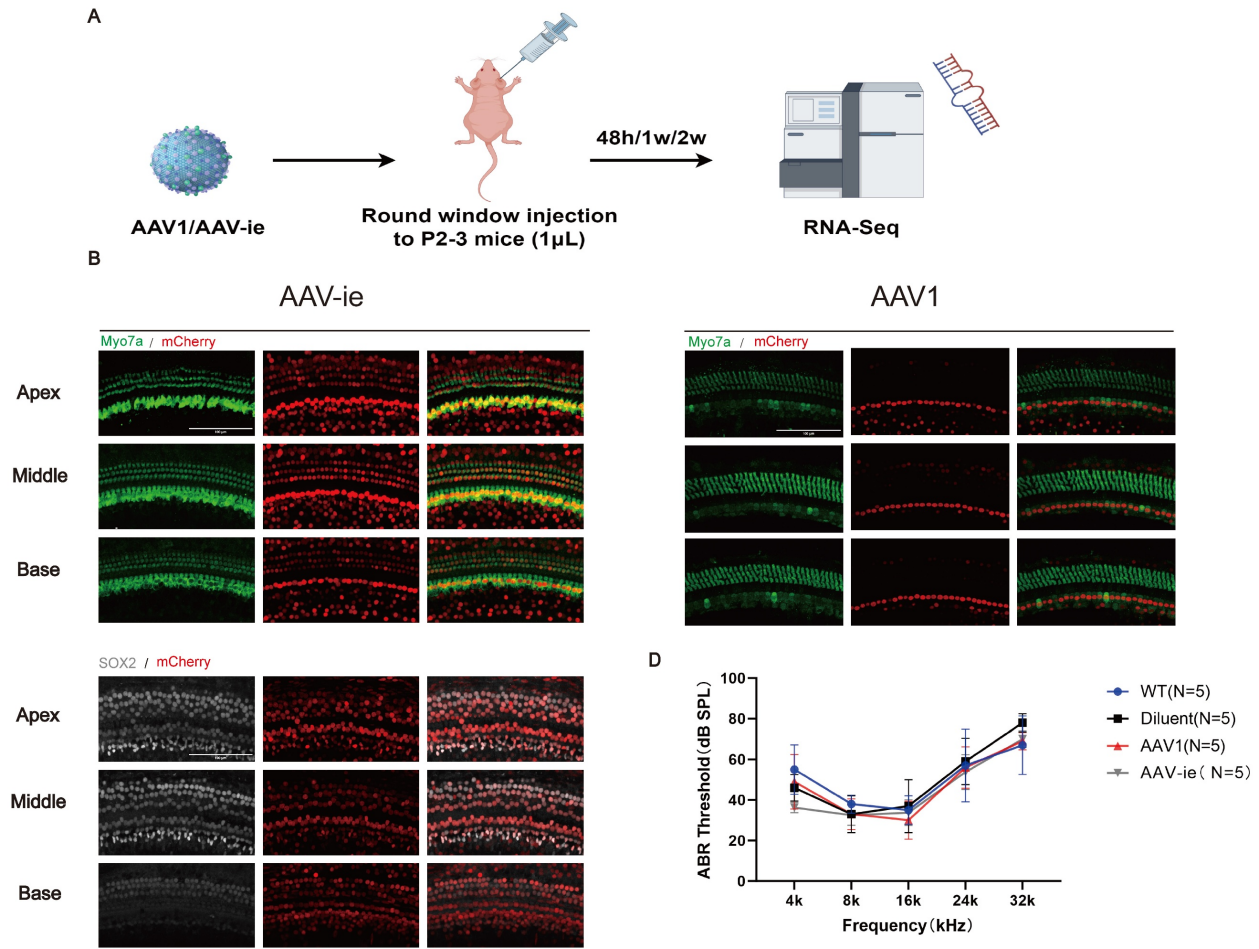
AAV1 and AAV-ie are safe and effective vectors for neonatal cochlear gene delivery. (A) Schematic timeline of intracochlear delivery: Postnatal day 2-3 (P2-3) mice received unilateral injections of AAV1 or AAV-ie via the RWM. Cochleae were harvested at 48 hours, 1 week, and 2 weeks post-injection for RNA sequencing. (B) Representative immunofluorescence images of cochlear sections 2 weeks post-injection. Hair cells were labeled with Myosin7a (green), and mCherry (red) indicates transduced hair cells. And transduced cells were predominantly localized to the organ of Corti, with minimal off-target expression in supporting cells (Sox2^+^). Scale bar: 100 μm. (C) Transduction efficiency of AAV1 and AAV-ie in inner hair cells (IHCs) and outer hair cells (OHCs) across apical, middle, and basal cochlear turns. (D) ABR thresholds measured at 4 weeks post-injection showed no significant differences in hearing sensitivity between AAV1-, AAV-ie-injected ears, and WT (wild-type mice without injection or dilute-injected ears).

Cochleae were harvested at 48 hours, 1 week, and 2 weeks post-injection (Figure 1A) (n = 3 per group/time point), flash-frozen in liquid nitrogen, and stored at -80 °C. To analyze cochlear transcriptomes, total RNA was isolated with TRIzol reagent and processed for bulk RNA-seq on the Illumina NovaSeq 6000 platform. The raw data are available under NCBI BioProject accession code PRJNA1314849 (https://www.ncbi.nlm.nih.gov/). Following sequencing, raw data quality was assessed by FastQC before genome alignment against the mm10 reference using STAR. Transcriptomic profiling revealed significant DEGs (DESeq2; |log2FC| ≥ 1, FDR < 0.05), which were subsequently characterized through functional annotation using ClusterProfiler for GO term and KEGG pathway analyses.

### 2.8 qRT-PCR validation of immune-related genes

Relative mRNA expression was quantified using qRT-PCR with SYBR Green PCR Master Mix. Following the addition of forward and reverse primers, the reaction mixtures were treated as follows: incubated at 95 °C for 10 minutes, then subjected to 40 cycles of 95 °C for 10 seconds and 58 °C for 10 seconds. A final melting curve analysis was added to verify the specificity of the PCR product. All experimental procedures were conducted in biological triplicate, and the data were analyzed utilizing the comparative cycle threshold (Ct) method. Gapdh served as the internal control, and the relative quantification was determined using the formula: Amount of target gene = 2-ΔCt, where ΔCt = Ct of target genes - Ct of Gapdh. The gene-specific primers are detailed in Supplementary Table 1.

### 2.9 Statistical Analysis

Data are presented as mean ± SD. Group comparisons employed two-way ANOVA with Tukey's post-hoc tests (GraphPad Prism v10). Statistical significance was set at *p* < 0.05.

## 3. Results

### 3.1 AAV1 and AAV-ie are safe and effective vectors for neonatal cochlear gene delivery

AAV1 is a well-characterized serotype approved for use in multiple clinical trials, including *OTOF* hearing loss gene therapy, and it can effectively transduce inner hair cells. And AAV-ie is engineered for enhanced tropism to cochlear cells, particularly for supporting cells (SCs) and hair cells (HCs). In this study, both AAVs were selected as representative vectors due to their distinct clinical and mechanistic profiles in auditory research. P2-3 mice received unilateral injections of AAV1 or AAV-ie via RWM. Cochleae were harvested for RNA sequencing at 48 hours, 1 week, and 2 weeks post-injection (Figure 1A).

To assess regional transduction patterns, whole-mount cochlear preparations from injected ears were analyzed (Figure 1B and C). Immunofluorescence analysis at 2 weeks post-injection revealed robust tdTomato expression in hair cells labeled by *Myosin7a* in AAV-ie group (Figure 1B). Transduced cells were predominantly localized to the organ of Corti, with highly expression in supporting cells (Sox2^+^) (Figure 1B). Consistent with prior reports, AAV1 exhibited strong tropism for IHCs but limited OHC transduction (Figure 1C). While AAV-ie achieved higher transduction rates for HCs and SCs (Figure 1B), as reported.

To evaluate possible hearing damage caused by AAV serotypes, ABR was assessed four weeks after vector administration. ABR assessments revealed no significant differences in hearing thresholds between AAV-injected ears and control groups (Figure 1D). These findings indicate that both AAV1 and AAV-ie enable safe and efficient gene transfer to inner ear cells and do not induce structural or functional harm to the auditory system.

### 3.2 Gene differential expression in cochlear post AAV1 /AAV-ie administration

**Figure 2:**
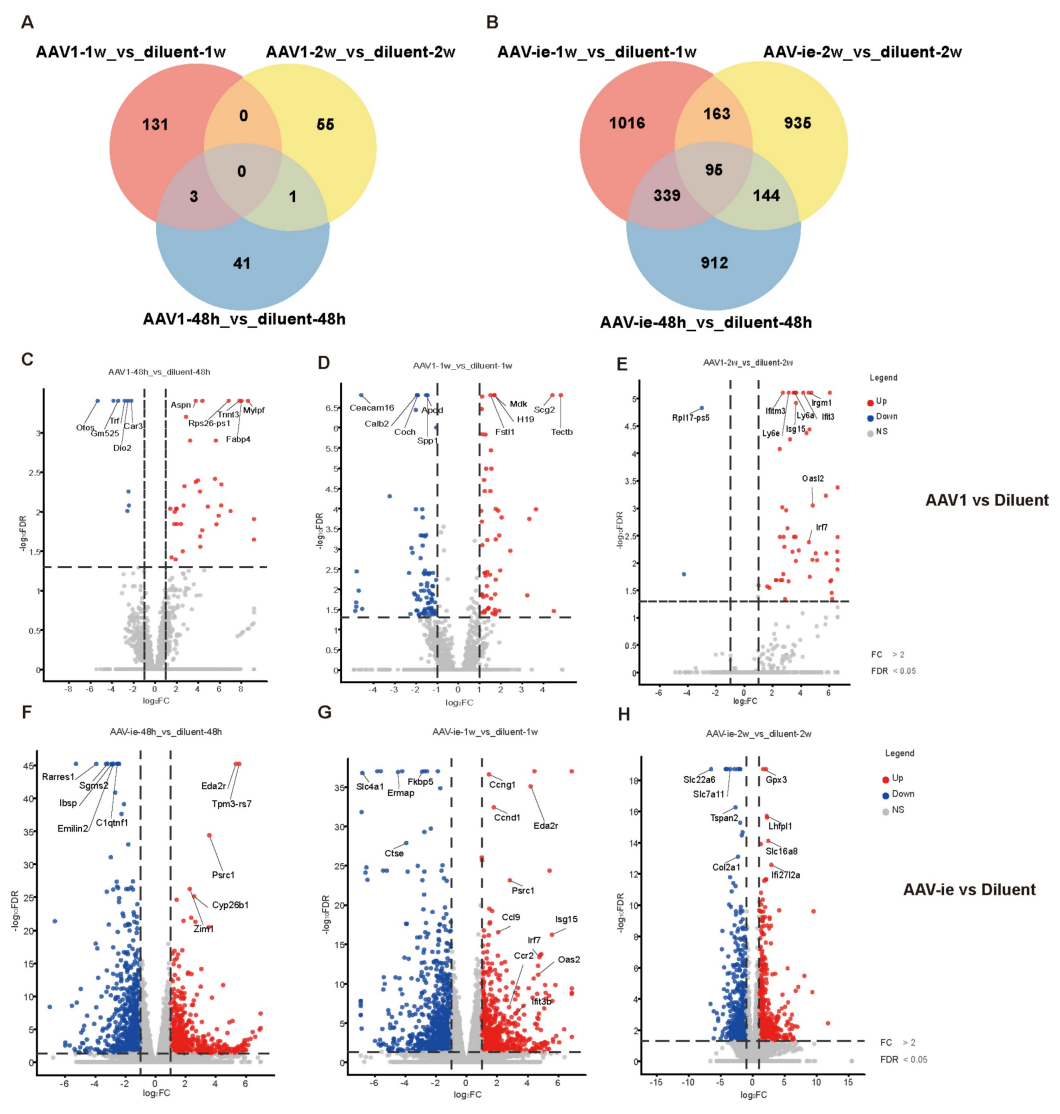
Comparative transcriptomic profiles of AAV1 and AAV-ie treatments across timepoints. Volcano plots display differentially expressed genes (DEGs) at (A-C) 48 h, 1 week, and 2 weeks post-AAV1 administration, and (D-F) corresponding time points for AAV-ie treatment. Red dots: upregulated genes (log2FC ≥ 1, FDR ≤ 0.05); blue dots: downregulated genes (log2FC ≤ -1, FDR ≤ 0.05); gray dots: non-significant genes.

To further study the immune response-related genes triggered by AAV, transcriptomic profiling was performed, revealing temporally resolved gene expression dynamics in response to the administration of AAV1 and AAV-ie, with a dilution solution injection serving as a control to elucidate the specific effects of AAV on the immune response. (Figure 2, The full list of DEGs identified in the analyses presented in Figure 2 have been attached as supplementary data 1). Compared to the dilution control group, the number of differentially expressed genes following AAV1 injection at 48 hours, 1 week, and 2 weeks was 45, 134, and 56, respectively. In contrast, the injection of AAV-ie resulted in significantly higher numbers of differentially expressed genes, with 1490, 1613, and 1337 detected at the same time points. Overall, AAV-ie transduction in the inner ear appears to exert a more pronounced effect on the overall transcriptomic expression (Figure 2A and B). The volcano plot data show that several immune-related molecules, such as *Ifitm3*, *Isg15*, and* Oasl2*, are significantly upregulated at 2 weeks post-injection with AAV-1, indicating their potential roles in the immune response triggered by AAV administration. While these genes exhibit noticeable upregulation just 1 week after AAV-ie injection, this early response may be triggered by robust transduction of AAV-ie. (Figure 2C-H). This suggests that there may be differences in the timing and intensity of the immune response elicited in the inner ear following AAV-1 and AAV-ie injections.

At the same time, we compared the gene expression differences between the injection group with the diluent and the wild-type mouse group at 48 hours, 1 week, and 2 weeks. We found that only a small number of genes showed significant differences (with 1 different gene at 48 hours, 3 different genes at 1 week, and 41 different genes at 2 weeks). This indicates that the injection procedure itself does not have a significant impact on the cochlea (Supplementary Figure 1).

### 3.3 Functional enrichment analysis of DEGs post AAV1 /AAV-ie administration

**Figure 3:**
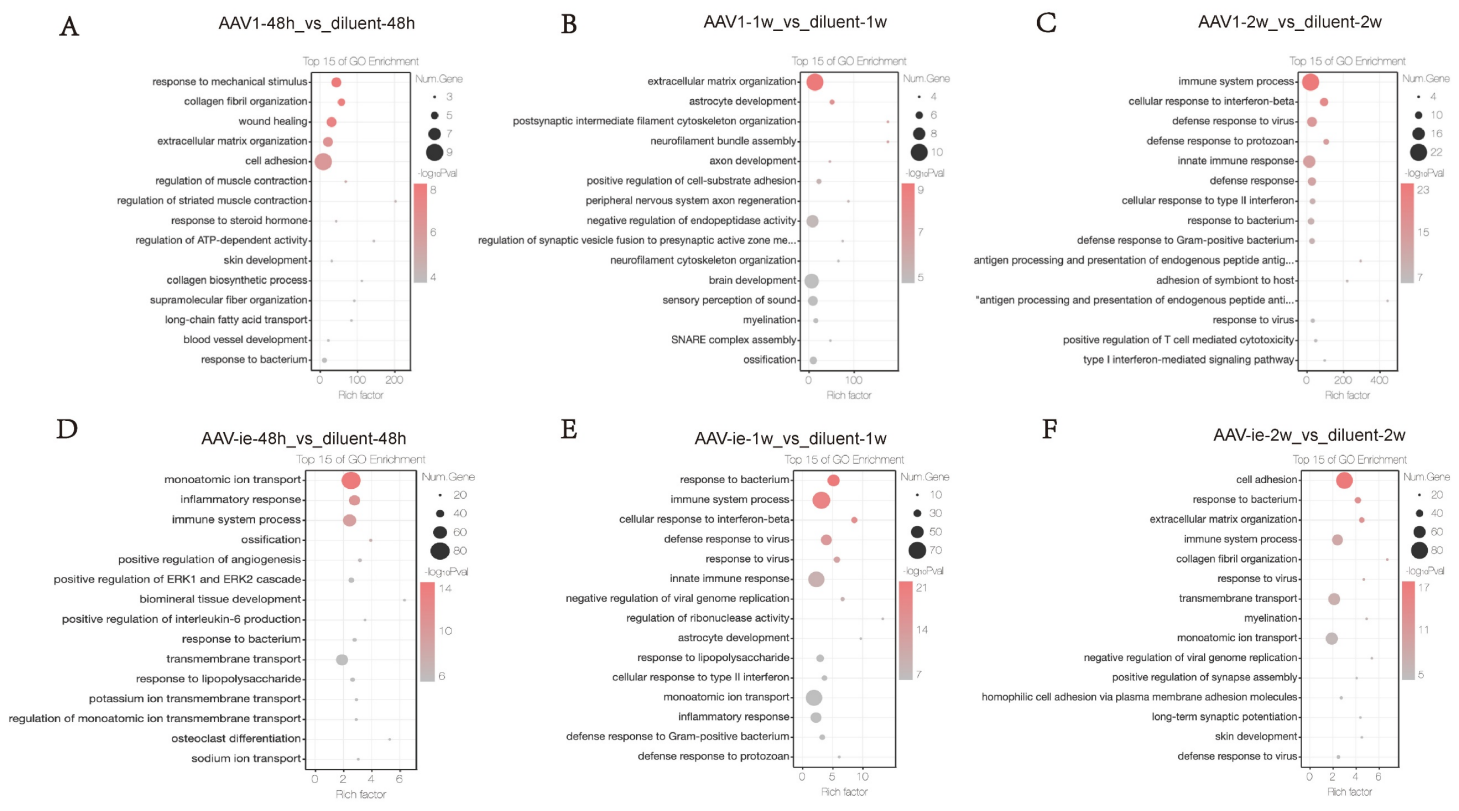
Gene ontology (GO) Analysis of differential gene expression between AAV administration and diluent treatment, (A-C) AAV1 transduction induces an immune response by week 2. In contrast, (D-F) AAV-ie administration triggers inflammation at 48 hours post-injection and shows an immune response as early as 1 week.

To further investigate the functional enrichment of DEGs between the AAV administration and diluent groups, GO analysis was conducted. No significant immune processes were detected at the 48-hour and 1-week time points (Figures 3A-B). In contrast, two weeks post-AAV1 administration, we observed a strong enrichment of gene groups related to immune processes, cellular responses to interferon-beta, defense responses to viruses, innate immune responses, and general defense responses (Figure 3C). In the context of AAV-ie injection, genes associated with inflammatory responses and immune processes were observed to be activated as early as 48 hours post-injection. At 1 week, immune-related processes such as cellular responses to interferon-beta, defense responses to viruses, and innate immune responses, which emerged 2 weeks after AAV1 injection, showed significant enrichment. However, by the 2-week post-injection, the enrichment of these gene groups had noticeably decreased (Figure 3D-F). These results suggest that AAV1 may take longer to elicit an adaptive immune response, potentially due to its mechanism of transduction and the type of immune cells it engages. AAV-ie may provoke a more rapid inflammatory response, possibly because of the vector properties or the immune recognition patterns it evokes. Overall, it seems that the timing of immune responses can vary significantly between different AAV protocols, highlighting unwanted immune responses.

### 3.4 DEGs associated with GO terms "response to virus" and "inflammatory response," and their confirmation

**Figure 4:**
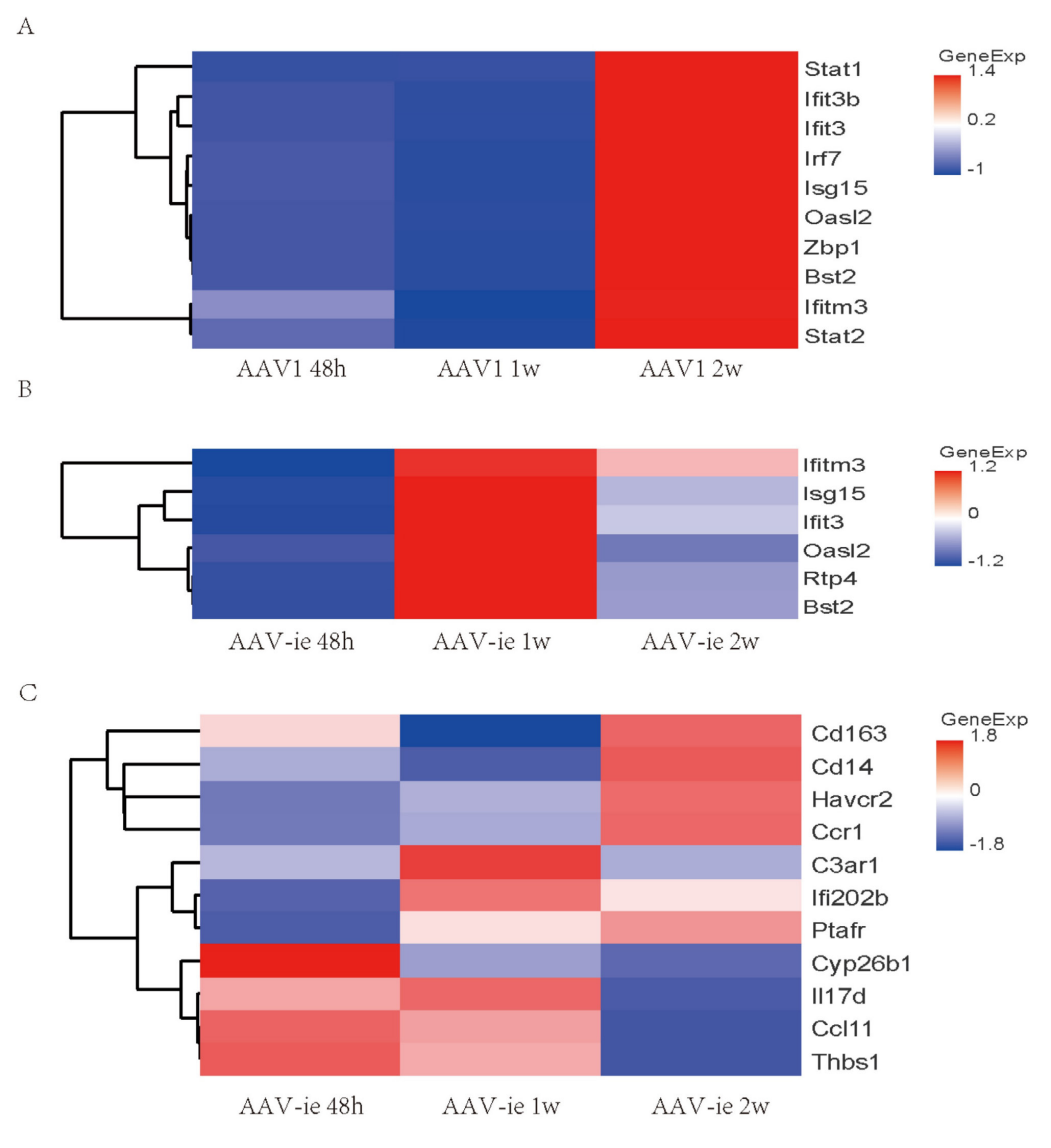
Heat map of select differential genes in the AAV-administered cochlea. (A) genes associated with the GO term “immune response to virus” in cochlea 48h, 1w, and 2w post AAV1 administration. (B) genes associated with the GO term “immune response” to virus in the cochlea 48h, 1w and 2w post AAV-ie administration. (C) genes associated with the GO term “inflammatory response" in the cochlea 48h, 1w and 2w post AAV-ie administration.

**Figure 5:**
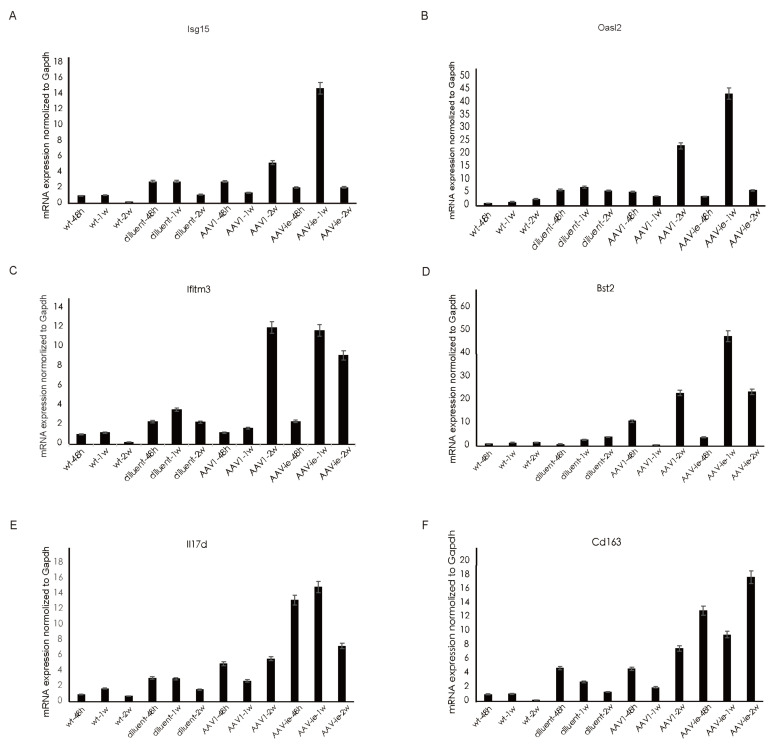
Validation of selected gene expression related to the GO terms "immune response to virus" and "inflammation" following administration of AAV-1 and AAV-ie. (A-D) relative expression of Isg15, Oasl2, ifitm3 and Bst2, which are related to the GO term "immune response to virus", (E-F) relative expression of IL17d and CD163, which are related to GO term "inflammatory response".

Consistent with the trend observed in GO analysis, genes related to the GO annotation "response to virus" showed a significant increase in expression 2 weeks after AAV1 injection (Figure 4A). In contrast, the expression of these genes peaked 1 week after AAV-ie injection and then significantly decreased by the 2-week mark (Figure 4B-C). We performed qRT-PCR validation on several of these genes, the results showed that relative expression of Isg15, Oasl2, ifitm3 and Bst2, which are related to the GO term "immune response to virus", were almost consistent with the transcriptome data. Among them, Bst2 shows a significant upregulation and reaches a peak one week after AAV-ie infection, as demonstrated by the transcriptome data. However, the data from two weeks later indicate that its expression level did not drop to a trough; instead, it is comparable to the data from the AAV-1 infection group at two weeks, showing slight deviation from the transcriptome data in Figure 4. This discrepancy may be due to variations in the qPCR data or individual differences among the mice. Overall, the expression trend—with an increase to a peak at one week followed by a clear decrease at two weeks—aligns with the transcriptome data (Figure 5). However, the situation with molecules related to the GO term “inflammatory response” is more complex. Some molecules, such as* IL-17d* and *Ccl11*, began to show upregulation in expression as early as 48 hours after AAV-ie injection, with expression levels receding by 2 weeks. The peak expression of *C3ar1* and *Ifi202b* occurred at 1 week, while molecules like *Cd163*, *Cd14*, and *Ccr1* showed significant upregulation only at 2 weeks (Figure 4C). Notably, there were no significant expression differences for these inflammatory response-related molecules following AAV-1 injection. Thus, it is evident that the changes in the transcriptome induced by AAV-ie injection in the inner ear are more complex.

## 4. Discussion

In recent years, gene therapy for hereditary hearing loss has made significant strides, particularly through AAV-mediated methods 2. A wealth of research has shown that gene replacement therapy using AAVs can significantly enhance auditory functioning in various animal models suffering from hereditary hearing loss 19, 20. Littermates receiving AAV vector injections showed negligible alterations in auditory sensitivity, suggesting AAVs' efficacy as viral carriers in safely delivering therapeutic genes to the mammalian inner ear. Additionally, the first human clinical trial for *OTOF*-related deafness showed remarkable success, using a dual-AAV vector system to deliver functional *OTOF* genes based on AAV1, and restore hearing in children with profound congenital deafness 5.

Although both AAV1 and Anc80L65 have demonstrated their safety in *OTOF* gene therapy 5, 6, recent studies indicate that different AAV capsid types can provoke varying immune responses in the inner ear. AAV2 and AAV2/7m8 transduction caused an increase in macrophage 3 days post-injection, the number of macrophages peaked at day 14 and declined by day 28 17. AAV9 provoked more significant cochlear inflammation than AAV1, marked by higher macrophage infiltration (F4/80+/ CD68+) and broader spatial distribution 16. These studies provide us with information on the changes in immune cells, primarily macrophages, following AAV infection in the inner ear, as well as the production of neutralizing antibodies. However, there has not yet been in-depth research on the changes at the gene expression level caused by AAV infection in the inner ear.

In this study, we aim to investigate whether there is a cellular stress response in the inner ear at the early stage of infection (48 hours) and the elicitation of immune responses at relatively later stages (1 week and 2 weeks) through changes in the transcriptome following AAV infection. Compared with the diluent treatment group, we did not observe fluctuations in gene expression related to cellular stress in AAV treatment at 48 hours. However, we observed differences in the expression of immune-related genes at the time points between AAV1 and AAV-ie. At 48 hours, AAV-1 transduction resulted in only 45 DEGs, primarily linked to damage repair processes, including responses to mechanical stimuli and wound healing. In contrast, immune-related genes began to appear at week 2 with a slight up-regulation. AAV-ie, on the other hand, initiated inflammation and immune responses as early as 48 hours, with the peak expression of immune-related genes occurring at one week. This observation correlates with the biodistribution patterns of the two capsids.

The changes in the transcriptome following AAV1/AAV-ie transduction help researchers to identify affected signaling pathways, contributing to the understanding of the biological effects mediated by AAV and its regulatory mechanisms. Genes associated with the ERK1 and ERK2 cascade showed an up-regulation accompanied by AAV-ie administration, which is worth further study afterward.

From the observations of AAV1 and AAV-ie transduction, it can be inferred that AAV capsids with high transduction efficiency in the inner ear may lead to stronger and faster immune and inflammatory responses 21. This finding is consistent with comparisons mentioned in other literature between AAV2/7m8 and AAV2, as well as between AAV9 and AAV1 22, 23. As the field of inner ear gene therapy progresses, newer and more efficient AAV capsids are expected to be applied in the treatment of hereditary hearing loss 21. Our research also provides some insights for the application of new capsids; as the transduction efficiency increases, the immune response induced by AAV gene delivery will correspondingly enhance 21. To prevent potential damage to inner ear cells, certain commonly used immunosuppressants, such as glucocorticoids and dexamethasone, may be effective options 24.

The alterations in the transcriptome following AAV1/AAV-ie transduction will aid researchers in identifying the affected signaling pathways in the inner ear during both short-term and long-term periods (1 week and 2 weeks) post-AAV injection into the cochlea. These results could enhance our understanding of the biological effects mediated by AAV in the cochlea, as well as the underlying regulatory mechanisms. Notably, after AAV-ie administration, there was a significant upregulation of genes associated with the ERK1 and ERK2 cascade reaction. Further investigation into the significantly impacted signaling pathways within this cascade is warranted. Furthermore, this study examined the changes in the cochlear transcriptome at intervals of 2 days, 1 week, and 2 weeks after AAV1/AAV-ie injection, the time-course analyses offer dynamic insights into how gene expression changes evolve following transduction, while also emphasizing cell type-specific responses, which are crucial for understanding the functions and interactions of various cell types within the inner ear. In summary, our findings present serotype-dependent differences and temporal correlation differences in the cochlear immune response at the transcriptomic level after AAV1/AAV-ie injection. This work provides insights into the significant advancements in inner ear biology and related therapeutic approaches.

## Supplementary Material

Supplementary figure and table.

Supplementary data 1 - figure 2 raw data.
