# Supplementary material for: Transcriptomic Profiling Reveals Divergent Immune Responses to AAV1 and AAV-ie in Mice Inner Ear
Source: Int J Med Sci. 2026 Jan 8;23(2):611–9. doi: 10.7150/ijms.121060 (PMC12825142; doi:10.7150/ijms.121060)
Supplement: Supplementary file 1 — Supplementary figure and table. [file ijmsv23p0611s1.pdf]

Supplementary data 1

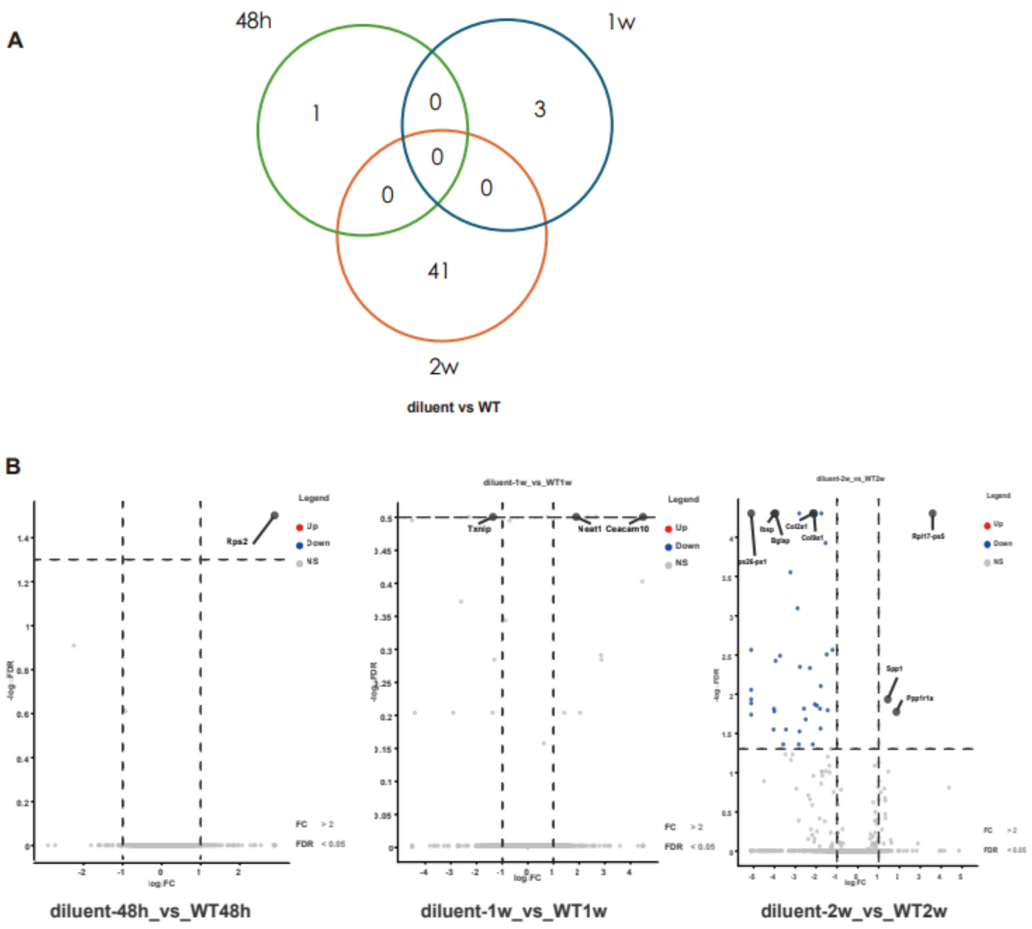

Supplementary data 1: Differentially expressed genes between diluent treatment and wild type mice.

A. Venn diagram and B. volcano plot show a few differentially expressed genes between diluent and wild type mice.

Supplementary Table 1

B. Isg15-F	TATGCTGCACCCACCAAGAG
Isg15-R	AAAGAGACGGAAGGCAGCTC
Oasl2-F	CGGGAACAGTGGATTGGACA
Oasl2-R	GGAGGAAAGGTCTCCAGCAC
Ifitm3-F	CAACATGCCCAGAGAGGTGT
Ifitm3-R	TCACGGAGTAGGCATAGGCT
Bst2-F	CCTTCAGCAAGGACTGGTCT
Bst2-R	TCTCGATCCCAGTAGACGGT
Il17d-F	ATGGGCAAGTGCAAGGTGTA
Il17d-R	TGGTGTAGTGTGGTGACCCT
Cd163-F	TGGGAATGACGACAGCCAAA
Cd163-R	ATGTCTTGGCCTTCCTGACG
Gapdh-F	CATCACTGCCACCCAGAAGACTG
Gapdh-R	ATGCCAGTGAGCTTCCCGTTCAG

Supplementary Table 1: Primers used in QPCR
